# Optimizing outcomes in intrauterine insemination: A narrative synthesis of two decades of clinical research (2000–2024)

**DOI:** 10.1002/ijgo.70847

**Published:** 2026-02-25

**Authors:** Kasuni Akalanka

**Affiliations:** ^1^ Rural Health Research Institute Charles Sturt University Orange New South Wales Australia

**Keywords:** body mass index, endometrial thickness, intrauterine insemination, intrauterine insemination success, luteal support, modifiable risk factors, slow‐release insemination, sperm parameters, total motile sperm count

## Abstract

Intrauterine insemination (IUI) is a widely used first‐line fertility treatment, yet its success rates vary considerably. This review synthesizes evidence from peer‐reviewed studies published between 2000 and 2024, including randomized controlled trials, cohort and case–control studies, as well as systematic and narrative reviews identified through structured searches of PubMed and Google Scholar, to examine the influence of patient characteristics, procedural variables, and environmental factors on IUI outcomes. Maternal age, body mass index (BMI), psychological stress, and nutritional status (insufficient intake of unsaturated fatty acids, vitamins [folate, B12, A, C, and E], minerals [nickel, zinc, and selenium]), strongly influence treatment success. Female‐specific factors, including the number of mature follicles, endometrial thickness (6–10 mm), and ovarian stimulation protocols are consistently associated with improved pregnancy rates. Male‐related determinants such as total motile sperm count, morphology, and DNA fragmentation index also play a significant role in influencing outcomes. In addition, procedural interventions, including slow‐release insemination, use of soft catheters, and luteal phase support, have demonstrated potential benefit in some studies; however, the evidence is drawn from small RCTs and observational studies, with heterogeneous effect sizes, and therefore findings should be interpreted cautiously. Environmental exposures, including tobacco, alcohol, and reproductive toxins, further modulate IUI efficacy, especially when combined with modifiable lifestyle factors. This narrative review highlights that IUI success is strongly influenced by specific clinical thresholds and patient characteristics. Higher pregnancy rates are observed when endometrial thickness is within 6–10 mm, post‐wash total motile sperm count is ≥5–10 million, and gonadotropin‐based stimulation protocols are used. Additionally, maintaining a healthy BMI, ensuring adequate micronutrient intake, and avoiding tobacco and alcohol exposure appear to support more favorable outcomes. These findings emphasize the importance of integrating evidence‐based clinical criteria with modifiable lifestyle optimization when selecting and managing IUI candidates.

**Abbreviations**
ART: assisted reproductive technology
BMI: body mass index
DFI: DNA fragmentation index
DGC: density gradient centrifugation
DHA: docosahexaenoic acid
ET: endometrial thickness
FSH: follicular stimulating hormone
ICSI: intracytoplasmic sperm injection
IUI: intra uterine insemination
IVF: invitro fertilization
MesH: medical subject headings
OHSS: ovarian hyperstimulation syndrome
PUFAs: polyunsaturated fatty acids
RCT: randomized controlled trials centrifugation
SU: swim‐up
TMC: total motile count

## 1 INTRODUCTION

Intrauterine insemination (IUI) is one of the widely used assisted reproductive technologies (ART), particularly for couples with unexplained infertility, mild male factor infertility, anovulation, or cervical factor infertility. The procedure involves placing processed, motile sperm directly into the uterine cavity to enhance the likelihood of fertilization by bypassing cervical barriers and optimizing sperm–oocyte proximity.^1^, ^2^ As a less invasive and more affordable alternative to in vitro fertilization (IVF), IUI is often the first‐line treatment in couples with favorable prognostic indicators.

Despite improvements in ovarian stimulation protocols and procedural techniques, IUI success rates remain modest and variable, ranging from 5% to 25% per cycle.^3^, ^4^ This heterogeneity is influenced by a combination of factors, including differences in cycle type (natural vs. stimulated), sperm source (partner vs. donor), female age, and semen quality, as well as underlying biological, clinical, and lifestyle determinants.^3^, ^5^, ^6^, ^7^ Common predictors of success include maternal age, total motile sperm count (TMSC), the number of mature follicles, endometrial thickness, and duration of infertility.^8^, ^9^

Emerging evidence has also emphasized the role of modifiable risk factors in influencing reproductive outcomes. Elevated body mass index (BMI) is associated with anovulation, impaired endometrial receptivity, and hormonal imbalances; however, its direct effect on IUI outcomes remains inconclusive.^10^, ^11^, ^12^, ^13^ Similarly, tobacco use and alcohol consumption negatively affect both male and female reproductive parameters, including sperm motility and ovarian reserve.^14^, ^15^, ^16^ Nutritional factors, particularly folate, zinc, selenium and antioxidant vitamins, are known to influence spermatogenesis and endometrial receptivity, serving as important preconception fertility markers. While these nutrients might indirectly contribute to improved chances of conception, direct evidence linking specific micronutrient deficiencies to IUI success rates remains limited and heterogeneous.^17^, ^18^

Psychological factors such as elevated stress and anxiety have been associated with reduced treatment adherence and lower clinical efficacy, although the underlying biological mechanisms remain poorly understood.^19^ Environmental and occupational exposures, including air pollution, pesticides, and endocrine‐disrupting chemicals, are also emerging as significant contributors to impaired reproductive outcomes and reduced IUI success rates.^20^, ^21^ In addition, clinical and procedural variables, such as the choice of ovarian stimulation protocol (gonadotropins vs. clomiphene), use of luteal phase support, timing of insemination, and insemination technique (slow‐release vs. bolus), have been shown to influence pregnancy outcomes.^22^, ^23^, ^24^

Given the multifactorial nature of IUI success and the heterogeneity across published studies, there is a clear need for an integrative synthesis to inform clinical decision‐making and individualized patient counseling. This review critically examines the biological, clinical, lifestyle, and environmental determinants of IUI success, with particular emphasis on modifiable factors and their potential to enhance treatment outcomes. The aim is to provide a comprehensive, evidence‐based framework to support personalized fertility care and improve reproductive success.

## 2 METHODOLOGY

### 2.1 Study design and selection criteria

Studies of various designs that evaluated factors associated with IUI success were thoroughly reviewed. Eligible studies included case studies, parallel group and crossover randomized controlled trials (RCTs), meta‐analyses, and retrospective observational studies. Studies of any design were included if they investigated patient‐related or cycle‐specific predictors of IUI outcomes, particularly the clinical pregnancy rate.

### 2.2 Search strategy

A comprehensive literature search was performed via PubMed and Google Scholar to identify relevant articles published between 20 January 2000 and 20 March 2024. The search strategy employed combinations of Medical Subject Headings (MeSH) terms and free‐text keywords, such as “intrauterine insemination,” “IUI,” “predictive factors,” “success rate,” “sperm parameters,” “total motile count,” “number of follicles,” “endometrial thickness,” “BMI,” “alcohol consumption,” “smoking,” “nutritional factors,” “maternal factors,” and “paternal factors.”

The search results were screened by title and abstract, and potentially eligible articles were reviewed in full text. The reference lists of the included articles were manually searched to identify any additional relevant studies.

### 2.3 Inclusion and exclusion criteria

Studies were included if they:

- Reported factors influencing IUI success rates
- Presented original data or meta‐analytic findings
- Were published in English between 2000 and 2024
- Focused on human subjects receiving IUI treatment.

Studies were excluded if they:

- Lacked relevant outcome measures (e.g., clinical pregnancy)
- Focused solely on donor insemination without analyzing patient‐specific predictors
- Were expert opinions, or editorials.

### 2.4 Data extraction and synthesis

Data extracted from each included study encompassed study design, population characteristics, IUI protocol details, and identified predictors of clinical pregnancy. A qualitative synthesis approach was used to summarize and compare key themes and patterns across studies. Where available, quantitative indicators such as odds ratios, confidence intervals, or correlation coefficients were presented descriptively to illustrate the strength and direction of associations without performing statistical pooling.

## 3 RESULTS

A total of 180 out of 1670 identified articles were reviewed, and 89 met the inclusion criteria for this review. The data were synthesized based on clinically relevant categories: patient‐related, male‐related, female‐related, procedural, and environmental factors. Table 1 provides a comprehensive overview of the extracted data, and the results are discussed according to each section.

**TABLE 1:** Summary of modifiable and non‐modifiable determinants associated with IUI success.

Determinant (units)	Optimal threshold/risk level	Impact on IUI outcome	Modifiability	References
BMI (female) (kg/m^2^)	18.5–24.9	Obesity linked to anovulation, poor endometrial receptivity	Yes	11, 12, 13, 29, 30, 31, 32, 33, 34, 35, 36, 37, 38
BMI (male) (kg/m^2^)	18.5–24.9	Obesity decreases sperm quality and motility	Yes	11, 12, 13, 39, 40, 41, 42, 43, 44, 45
Smoking	Abstinence	Reduces oocyte and sperm quality; halves success rates	Yes	14, 18, 19, 20
Alcohol intake (none, mild to moderate)	Moderate to none	Heavy use negatively affects reproductive parameters	Yes	22, 23, 24, 25
Nutritional status	Balanced diet with vitamins and minerals	Deficiencies impair spermatogenesis and endometrial health	Yes	15, 16, 17
Psychological stress	Low levels of stress	High stress affects compliance, possibly hormonal balance	Yes	27, 28
Environmental toxins (exposure)	Minimal exposure	Linked to reduced sperm and egg quality	Partial	20, 21
Ovarian stimulation protocol	Gonadotropins preferred	Improves pregnancy rates over clomiphene; monitor for OHSS	Yes (clinical choice)	^23^
Timing of insemination (hours post hCG)	36 h post‐hCG	Optimal window for fertilization	Yes (clinical strategy)	2, 23, 76
Luteal phase support	Progesterone supplementation, 200–400 mg/day ±50 mg	Improves endometrial receptivity and implantation rates	Yes (clinical strategy)	^24^
Cycle count	≤4 cycles	Diminishing returns beyond 4–6 cycles	Yes (clinical strategy)	8, 23
Number of mature follicles (count)	2–3 follicles	Higher pregnancy rates; >3 increases multiple gestation risk	Yes (clinical strategy)	8, 10, 18, 71, 72
Endometrial thickness (ET) (mm)	7.8 ± 1.2	Associated with improved implantation and clinical pregnancy	Yes (medical/lifestyle)	10, 32, 75
Total motile sperm count (TMSC)	≥5 million/(8.6 ± 3.1 million)	Strong positive predictor; <2 million reduces success	Yes (medical/lifestyle)	7, 8, 9, 38, 49, 50, 51, 54, 55, 61
Sperm morphology	≥4% normal forms/(5.1 ± 1.4%)	Associated with higher pregnancy rates	Partial (lifestyle)	9, 10
Progressive sperm motility	≥30%/38 ± 6.5%	Improves fertilization rates	Partial (lifestyle)	^9^
DNA fragmentation index (DFI)	<30%	High DFI linked to miscarriages and poor outcomes	Partial (lifestyle)	9, 64, 65, 66, 67, 68, 69
Duration of infertility (years)	<3	Longer duration linked with lower success	Partial	7, 8
Maternal age (years)	<35	Significant decline in pregnancy rates beyond age 35	No	2, 3, 6, 8

*Note*: Thresholds reflect ranges commonly reported across multiple observational and cohort studies evaluating predictors of IUI success. Values are intended to guide clinical interpretation rather than serve as absolute eligibility criteria. Modifiability refers to the extent to which each determinant can be influenced through lifestyle, medical, or clinical intervention.

Thresholds and impact levels are based on evidence from multiple studies and represent the most commonly cited parameters associated with IUI success. Clinical application should be individualized based on patient characteristics and treatment protocols.

### 3.1 Body mass index

Overweight and obesity are well‐established factors associated with subfertility in both women and men. In women, an elevated BMI has been linked to menstrual irregularities, oligo‐ovulation, and other reproductive disturbances.^4^, ^5^ While some studies suggest a positive relationship between higher BMI and pregnancy rates,^6^, ^7^ others report no significant association or even a detrimental effect, particularly when BMI exceeds 25–30 kg/m^2^.^8^, ^10^ In men, both overweight and obesity have been associated with reduced fertility potential. An increased BMI correlates with impaired semen quality, including lower ejaculate volume, decreased sperm concentration, reduced TMSC, and abnormal morphology. These negative effects are particularly evident in men with a BMI ≥30 kg/m^2^.^11^

### 3.2 Effect on ovulation induction response in intrauterine insemination cycles

In the context of IUI treatment, obese women often require higher doses of gonadotropins to achieve adequate follicular stimulation,^7^, ^11^ potentially influencing treatment success. This suggests that excess body weight might negatively influence ovarian responsiveness during IUI cycles, resulting in suboptimal follicular development, prolonged stimulation duration, and higher medication burden. Further, impaired endocrine function, altered follicular environment, and insulin resistance frequently observed in women with elevated BMI might further compromise ovarian response, making it more challenging to achieve optimal cycle parameters required for successful IUI outcomes.

### 3.3 Effect on intrauterine insemination pregnancy outcomes

The relationship between BMI and IUI pregnancy rates remains inconsistent throughout the literature. Some reports indicate that a BMI of 25–29.99 kg/m^2^ does not negatively affect live birth rates,^8^, ^10^ yet other studies highlight reduced pregnancy rates among women with BMI >25 kg/m^2^. For example, a retrospective analysis revealed a significantly lower pregnancy rate of 1.6% in women with BMI >25 kg/m^2^ compared to 7.2% in those with BMI <25 kg/m^2^.^7^, ^11^, ^12^ Nonetheless, some investigations have not demonstrated a clear link between elevated maternal BMI and IUI pregnancy rates,^13^ and others report no significant impact of maternal or paternal BMI on IUI outcomes.^14^ Further, obesity has been linked to increased risk of biochemical pregnancy following IUI.^11^, ^12^

Overall, although findings vary across studies, the prevailing evidence suggests that excess body weight impairs fertility outcomes in both women and men, potentially affecting natural conception and ART such as IUI. These observations underscore the importance of considering BMI in fertility management.

### 3.4 Nutritional parameters

Nutritional factors directly influence reproductive health and consequently affect the success of IUI. Dietary patterns characterized by higher intake of polyunsaturated fatty acids (PUFAs), folate, fiber, starch, and adherence to a Mediterranean‐style diet have been associated with improved fertility outcomes in women, whereas trans‐saturated fatty acids have been linked to adverse reproductive effects, including endometriosis and ovulatory dysfunction.^15^ Studies have indicated that several micronutrients, including zinc, selenium, folate, and vitamins B12, A, E, and C, as well as trace elements such as nickel and manganese, are critical for optimal spermatogenesis. In addition to micronutrients, macronutrients substantially influence male reproductive parameters. Docosahexaenoic acid (DHA), a PUFA, has been shown to positively affect sperm morphology and function; tuna oil, which is rich in DHA, serves as a valuable supplement. Additionally, arginine has been associated with improved sperm count and motility and can be sourced from seafood, watermelon juice, nuts, meats, and soy proteins. Collectively, these nutrients have significant effects on spermatogenesis, sperm concentration, motility, morphology, and overall semen quality.^16^ However, direct evidence linking specific micronutrient intake to IUI pregnancy rates remains limited, with most available studies reporting associations through intermediate fertility markers rather than IUI‐specific clinical outcomes. Although the role of vitamin D in fertility has been extensively studied, a recent cross‐sectional study indicated that vitamin D levels appear not to significantly influence IUI success rates.^17^

A nutritionally balanced diet, rich in both macro‐ and micronutrients, not only supports overall health but also enhances reproductive function. Further, diet serves as a key modulator of the gut microbiome, which has been shown to influence metabolic, hormonal, and reproductive health in women.^10^ Therefore, regular consumption of nutrient‐rich foods, including fish, shellfish, meat, poultry, nuts, beans, seeds, carrots, pumpkins, and a wide variety of fruits and vegetables, is likely to support metabolic, reproductive health and contribute to better fertility outcomes.

### 3.5 Smoking and alcohol consumption

Abstinence from smoking and alcohol prior to and throughout IUI treatment has been associated with improved reproductive parameters, thereby potentially enhancing IUI success. It has been reported that smoking in both partners reduces the success rate of IUI. Therefore, stopping or at least reducing smoking is recommended to increase the likelihood of IUI success.^14^ Smokers need higher doses of gonadotropins than nonsmokers do to obtain successful results from IUI,^19^ and adverse effects of male smoking have been reported.^20^ The underlying mechanism is presumed to involve a toxic effect of various tobacco chemicals on follicular development, gamete mutagenesis, or inhibition of granulose cell aromatase. This, in turn, leads to accelerated follicular depletion, reduced fecundity, increased miscarriages, and accelerated onset of menopause.^21^ Tobacco use, whether smoked or chewed, poses immediate and long‐term health risks, including carcinogenic effects. Nicotine, a highly addictive compound, can cause withdrawal symptoms such as anxiety and irritability. Reducing both active and passive tobacco exposure is associated with improved fertility and better pregnancy outcomes.

Evidence on lifestyle factors remains inconsistent. While some observational studies have reported that moderate consumption of coffee, tea, or alcohol is associated with higher pregnancy and live birth rates, others have shown no significant effect on IUI outcomes. These findings should therefore be interpreted as hypothesis‐generating, and further investigation warranted to clarify potential confounding and underlying mechanisms.^22^, ^23^

The evidence indicates that modifying male habits and work conditions can improve IUI outcomes, as studies report avoiding smoking, alcohol, and heat exposure before and during treatment increases success rates.^24^ While most studies have shown reduced sperm quality, concentration, motility, and morphology among smokers, a definitive causal link to infertility is unclear. Nevertheless, male smoking is associated with significantly lower clinical pregnancy rates.^25^ Although no strong link has been found between low‐to‐moderate alcohol intake or binge drinking and IUI success, further research is needed to confirm these findings.^26^

### 3.6 Anxiety level

Fertility challenges have a substantial psychological impact. Dealing with fertility problems negatively affects the psychological well‐being of the majority of couples. Treatment options are reportedly associated with psychological symptoms such as anxiety, depression, and irritability.^27^ Ovulation induction agents used in IUI protocols (e.g., gonadotropins, selective estrogen receptor modulators, and aromatase inhibitors) might further contribute to anxiety, irritability, and depressive symptoms through central effects on the hypothalamic–pituitary axis.^12^

The evidence linking psychological distress directly to IUI pregnancy outcomes, however, remains mixed. Some studies have not demonstrated a significant association between anxiety levels and clinical pregnancy rates following IUI.^28^ Nevertheless, biological and mechanistic data indicate that elevated stress might adversely influence fertility potential by increasing cortisol levels, disrupting gonadotropin‐releasing hormone pulsatility, impairing luteinizing hormone/follicular stimulating hormone (FSH) secretion, and reducing endometrial receptivity and implantation potential.^11^, ^13^ Stress‐related immune dysregulation and pro‐inflammatory cytokine activation might further compromise reproductive physiology.^14^

Therefore, while clinical evidence remains inconsistent, the biological plausibility of stress‐related impairment in reproductive function supports the importance of psychological support and stress management prior to and during treatment. Reducing stress might improve emotional coping, reduce treatment burden, and help decrease the number of cycles required to achieve pregnancy, potentially reducing the need for escalation to more invasive ART procedures.^11^, ^15^

## 4 SOCIODEMOGRAPHIC FACTORS

### 4.1 Age of women

Female age was consistently reported as the strongest predictor of IUI success. Continuing pregnancy rates were shown to be 38.5% for women <30 years versus 12.5% for those >40 years of age, and twin pregnancy rates also decrease significantly after 30 years.^29^ One report indicated that pregnancy rates for women between 19 and 40 years were approximately 10% and declined sharply beyond 40 years.^30^ Multiple studies confirm this negative correlation, demonstrating that IUI success declines as maternal age increases.^31^, ^32^ According to Patsama et al. (2015), the biochemical pregnancy rate decreased from 27.6% in women <35 years to 12.8% in women aged 35–40 years and 7.1% for those >40 years.^33^ Other studies similarly reported marked reductions in per‐cycle pregnancy rates in women ≥40 years (4.1%–7%) compared with 13.7–17% in women <40 years,^36^, ^37^ and one study further reported pregnancy rates of 11.1%–18.9% per cycle in women under 40 years, 4.7% in women aged 40–45 years, and 0.5% in women >45 years.^38^ A prospective cohort study reported a steep decline after 37 years and recommended consideration of earlier transition to IVF in older women to avoid repeated failed IUI attempts.^16^ Consistent with this, American Society for Reproductive Medicine guidelines indicate that IUI effectiveness is significantly limited in women over 40 years, and alternative ART strategies should be prioritized.^17^ The biological mechanism underlying this decline is attributed to a progressive reduction in oocyte quantity and quality, higher rates of aneuploidy, and diminished endometrial receptivity with advancing age.^18^ Collectively, these findings underscore the strong age dependency of IUI outcomes and highlight the need for timely fertility planning and age‐appropriate counseling, particularly among couples postponing childbearing.^29^, ^36^

### 4.2 Male age

Increasing paternal age, particularly when it exceeds 40 years, has an impact on reproductive outcomes, such as an increased incidence of premature birth,^39^ abortions,^40^ autism spectrum disorders,^41^ and infertility.^42^ There was no effect of paternal age on pregnancy or miscarriage rates among men aged 25–56 years (mean male age 34.3 years) who did not have severe male factor infertility.^43^ Compared with men aged <30 years, men aged >35 years have a decreased pregnancy rate.^44^ In contrast, another study demonstrated that age does not appear to greatly affect the pregnancy rate of male partners, although after the age of 50, there are more sperm abnormalities.^45^ Advancing paternal age has also been associated with increased sperm DNA fragmentation and higher levels of sperm aneuploidy, which might negatively influence reproductive potential and warrants consideration during counseling. Therefore, as subfertility is increasingly prevalent in modern societies, couples should be advised to avoid unnecessary delays in childbearing where possible, as age‐related reproductive decline is a modifiable factor only when childbearing postponement is elective.

## 5 MALE FACTORS

### 5.1 Sperm preparation techniques

Sperm preparation plays a critical role in IUI by isolating highly motile, morphologically normal sperm and eliminating seminal plasma, debris, and immotile or apoptotic spermatozoa. Among the most commonly used methods are density gradient centrifugation (DGC) and the swim‐up (SU) technique. DGC is widely preferred for its ability to yield a high concentration of motile sperm while effectively removing reactive oxygen species‐generating cells, which compromise fertilization potential.^42^ In a comparative study, superior sperm recovery and improved pregnancy outcomes with DGC, particularly in oligospermic samples, compared to the swim‐up method are reported^20^, ^43^ The SU technique, although less complex, remains effective in normozoospermic cases and offers a cost‐efficient alternative with acceptable clinical results.^44^ In addition, emerging techniques such as hyaluronic acid binding and magnetic‐activated cell sorting have shown promise in enhancing sperm selection; however, their routine use in IUI requires further validation through larger clinical trials.^45^

In the post‐COVID era, a notable shift has occurred in semen collection practices, with many fertility clinics adopting at‐home semen collection protocols to reduce in‐clinic exposure and improve patient comfort. While home collection offers convenience, concerns remain regarding the potential impact of extended transport time, suboptimal temperature control, and delays in sample processing on sperm quality parameters, including motility and viability.^46^ Studies have shown that delayed processing and improper transport conditions can negatively affect sperm function, ultimately reducing IUI success rates.^23^, ^47^ Recent evidence suggests that semen collected at home can retain acceptable quality if transported to the laboratory within approximately 1 h, with some data indicating that semen parameters might remain stable even when transport time extends up to 1.5–2 h.^48^ Therefore, minimizing transport time remains critical, as prolonged intervals between collection and processing increase the risk of motility decline and suboptimal insemination outcomes.

Additionally, the choice between using fresh and frozen sperm is another critical factor influencing treatment outcomes. Although frozen–thawed sperm is routinely used in donor insemination and offers logistical flexibility, it is associated with reduced motility and viability due to cryodamage during the freeze–thaw process.^24^, ^48^ Several studies report lower pregnancy rates with frozen sperm compared to fresh ejaculates, particularly in the context of IUI, although differences are less pronounced in IVF and ICSI settings.^25^, ^49^, ^50^ Therefore, both semen collection logistics and sperm preservation methods must be carefully optimized when designing protocols for IUI and other ART procedures. Tailoring sperm preparation techniques to semen quality significantly improves IUI outcomes, especially in cases involving male factor infertility.

### 5.2 Semen volume and sperm count

Semen parameters play a pivotal role in determining the likelihood of success with IUI. However, the volume of inseminated semen has not been significantly associated with pregnancy rates.^46^ The total sperm count within a processed specimen is consistently identified as a critical determinant of IUI outcomes.^47^ Among semen parameters, total progressive motile sperm count demonstrates the strongest association with IUI success, surpassing sperm concentration per milliliter in predictive value.

Several studies support the use of post‐wash TMSC of ≥10 million as a threshold for optimizing IUI success,^48^, ^49^ particularly in controlled ovarian hyperstimulation cycles for treating male subfertility.^50^ Similarly, post‐wash sperm count has emerged as a significant predictor, with high motility levels consistently linked to better clinical outcomes.^32^ The chance of achieving pregnancy diminishes notably when motile sperm counts fall below 5 million, especially among women with prolonged infertility exceeding 10 years.^30^, ^51^

Although motile sperm count is often emphasized, the evidence regarding total sperm count alone remains inconclusive. One study reported comparable pregnancy rates between individuals with normal (15.8%) and abnormal (15.2%) sperm counts; however, the abortion rate was substantially higher among those with abnormal counts (71.4% vs. 38.8%).^52^ Similarly, a retrospective analysis found no statistically significant association between sperm count and IUI success.^53^

Reported threshold values for TMSC in IUI outcomes vary widely across the literature. While some studies suggest successful pregnancies occur with TMSC as low as 0.3–1 million,^54^, ^55^ others report no live births when pre‐wash TMSC falls below 2 million.^56^ Despite occasional favorable outcomes in cycles with TMSC under 10 million,^48^, ^50^, ^58^ several studies consistently identify a minimum threshold of 5 million as a more reliable predictor of success.^29^, ^57^, ^59^ Post‐wash TMSC thresholds and the corresponding clinical pregnancy rate per IUI cycle (Table 2) indicates that a total TMSC exceeding 10 million is a significant predictor of IUI success.

**TABLE 2:** Post‐wash TMSC thresholds and corresponding CPR per IUI cycle.

Post‐wash TMSC category	Approximate CPR per cycle	Notes
<1 million	<2%	Very poor prognosis; IUI rarely beneficial; IVF/ICSI should be prioritized
1–4 million	Approximately 4%–8%	Low probability of success; IUI might be attempted but limited utility
5–9 million	Approximately 8%–12%	Moderate success range; acceptable IUI prognosis in appropriately selected couples
≥10 million	≥15%	Best expected outcomes; most favorable TMSC threshold for IUI

*Note*: Values represent approximate pooled ranges reported across multiple observational studies and clinical cohorts. Thresholds are intended to support clinical decision‐making rather than replace individualized assessment.

Abbreviations: CPR, clinical pregnancy rate; IUI, intrauterine insemination; IVF, in vitro fertilization; TMSC, total motile sperm count.

Importantly, lifestyle and environmental factors can significantly affect semen volume and motility. Factors such as physical inactivity, smoking, alcohol consumption, exogenous steroid use, and certain comorbidities have been linked to decreased sperm quality. Interventions focusing on lifestyle modifications, nutritional optimization, and targeted clinical management have been shown to enhance sperm function, potentially increasing the success rates of IUI and other ART.^26^, ^27^

### 5.3 Post‐wash sperm count

A post‐wash TMSC threshold of approximately 1 million has been reported across multiple studies as the minimum level at which pregnancy is still possible, with the lowest successful post‐wash count documented at 0.8 million.^29^, ^34^, ^60^ Several studies indicate that pregnancy rates do not substantially increase when post‐wash counts exceed 4 million, supporting approximately 1 million as an important minimum threshold in selected couples, particularly those with ovulatory dysfunction and cervical factor infertility.^29^

In addition, evidence consistently shows that TMSC is a key determinant of IUI success. Pregnancy rates decline when inseminated TMSC is <2 million,^38^ and a post‐wash TMSC ≥5 million has been strongly associated with higher success rates.^61^ One study reported optimal pregnancy rates with a TMSC threshold of 5 million combined with a post‐wash count of 1 million, particularly in ovulatory dysfunction and cervical factor infertility cases,^11^ as shown in Table 2. Overall, these findings support the clinical utility of both the minimum post‐wash count (approximately 1 million) and higher progressive post‐wash TMSC thresholds (≥5 million) in guiding IUI suitability and counseling.

### 5.4 Sperm morphology

Abnormal sperm morphology has been associated with poorer reproductive outcomes, including higher miscarriage risk. In one study using WHO 5th edition morphology criteria, patients with normal morphology demonstrated a pregnancy rate of 16.9% and an abortion rate of 35.6%, whereas those with abnormal morphology showed a reduced pregnancy rate of 15.0% and a higher abortion rate of 46.4%.^52^ These findings suggest a clear correlation between sperm morphology and both pregnancy and abortion rates.

Another study that assessed morphology using strict Kruger criteria also reported significant differences in normal sperm morphology between couples who achieved pregnancy and those who did not, despite comparable age and infertility duration, both before and after sperm processing.^53^ These findings suggest that morphology, whether assessed using WHO 5th edition or Kruger strict criteria, remains an important predictive parameter in IUI outcomes.^53^

### 5.5 Sperm concentration

Sperm concentration, defined as the number of sperm per milliliter, has traditionally been considered an important semen parameter; however, current evidence indicates that concentration alone is less predictive of IUI success than progressive post‐wash TMSC. Several studies have reported no significant independent association between sperm concentration and IUI pregnancy rates.^47^ Similarly, Findeklee et al. reported that sperm motility, concentration, and density did not influence IUI pregnancy outcomes.^63^ Noujua‐Huttunen et al. also found that the sperm concentration and density did not significantly influence IUI outcomes.^36^ However, studies have consistently demonstrated poorer outcomes when total progressive motile sperm count is <5 million post‐wash, reinforcing that TMSC is more clinically relevant than raw concentration alone.^62^ Therefore, in cases of male factor infertility where progressive TMSC remains persistently low and pregnancy is not achieved after approximately three IUI cycles, couples might benefit from counseling regarding transition to alternative ART options.^62^

### 5.6 DNA fragmentation index

The DNA fragmentation index (DFI) is a method used to assess DNA abnormalities in sperm. A DFI >30% is considered indicative of abnormal DNA integrity.^64^ Higher DFI levels have been positively correlated with increased pregnancy loss rates, with reported loss rates of 27.3% for high DFI, 14.6% for moderate DFI, and 4.9% for low DFI.^65^ Studies have reported lower biochemical pregnancy and delivery rates among couples with a DFI >30% than among those with a DFI ≤30%.^66^

Sperm DNA fragmentation has been associated with challenges at conception, prolonged time to pregnancy, poor outcomes following stimulated IUI, and impaired embryo development. These findings suggest that sperm DNA damage can have significant implications for reproductive outcomes.^66^, ^67^, ^68^, ^69^ However, other studies have reported no significant differences in pregnancy outcomes on the basis of the DFI.^4^, ^11^ In cases of significant DNA damage, alternative in vitro fertilization techniques are more appropriate.

Environmental and lifestyle factors also influence sperm DNA integrity. Notable contributors include exposure to radiation and heat, airborne pollutants, sexually transmitted infections, certain chemical agents and drugs, and being overweight or obese.^70^ Therefore, addressing modifiable risk factors might help optimize DNA integrity and improve outcomes. For men with persistently elevated DFI, clinical pathways might include assessment for correctable causes (e.g., varicocele), targeted lifestyle optimization, and cautious consideration of antioxidant therapy. If high DFI persists despite intervention and multiple unsuccessful IUI cycles, transitioning to IVF/ICSI might be warranted. Recent meta‐analytic evidence further indicates that interventions to reduce DFI vary in effectiveness, with varicocelectomy showing the most substantial and sustained improvement at 6 months, FSH therapy demonstrating potential benefit, antioxidant effects remaining modest and inconsistent, and lifestyle modification requiring further high‐quality studies before firm recommendations can be established.^55^, ^71^

## 6 MATERNAL FACTORS

### 6.1 Number of follicles

The number of follicles has a linear relationship with pregnancy rate, and increasing follicle numbers are associated with higher IUI success.^18^, ^32^, ^55^, ^71^ A retrospective cross‐sectional study also demonstrated that the duration of infertility had a highly significant impact on pregnancy rate and confirmed a positive correlation between the number of preovulatory follicles and pregnancy outcomes.^32^ However, this benefit must be considered alongside clinical risk. When endometriosis or tubal factor infertility contributes to subfertility, these conditions negatively influence IUI pregnancy rates.^18^

Multifollicular development, especially in cycles stimulated with gonadotropins, increases the risk of multiple gestations, including twins and higher‐order multiples, which in turn increases the likelihood of obstetric complications such as preterm birth and low birth weight.^28^ Additionally, the risk of ovarian hyperstimulation syndrome, although less frequent in IUI compared with IVF, rises when excessive follicular development occurs.^29^, ^74^ Therefore, while increasing follicle numbers improves pregnancy probability, this must be balanced against safety considerations. Many centers adopt a conservative policy for young women (<35 years), recommending cycle cancellation or conversion to IVF if ≥3 mature follicles are present, while slightly higher follicle thresholds might be acceptable in older women due to reduced multiple pregnancy risk.

Further, luteal phase support has been shown to improve outcomes in stimulated IUI cycles. The use of vaginal progesterone gel or micronized progesterone significantly increases clinical pregnancy rates and can positively influence live birth rates, particularly when gonadotropins are used for ovulation induction.^72^ Optimizing follicle development must, therefore, involve careful age‐based thresholds, stimulation dose adjustments, and meticulous monitoring to maximize benefit while minimizing maternal and fetal risks.

### 6.2 Endometrial thickness

Endometrial thickness (ET) is an important marker of endometrial receptivity in IUI cycles. Evidence suggests that ET below approximately 6 mm is associated with reduced pregnancy rates, whereas within the range of 6–10 mm, pregnancy likelihood appears to be more favorable.^31^, ^32^, ^75^ A secondary analysis of IUI cycles for unexplained subfertility also demonstrated that although the mean ET was 7.2 ± 1.8 mm, differences between pregnant and non‐pregnant groups within this range were small, and the association between ET and ongoing pregnancy was not statistically significant.^73^, ^74^ This aligns with meta‐analytic findings indicating that once ET exceeds approximately 6 mm, variations within 6–10 mm have limited predictive value, while data beyond 10 mm remain mixed and inconclusive. Age‐related changes in ET have also been described, with thinner endometrium more frequently observed in older women.^75^ Lifestyle and nutritional optimization, such as diets enriched with vitamin E and arginine, might support endometrial perfusion and hormonal regulation, potentially improving ET.^76^ Overall, ET <6 mm might signal poorer prognosis, while values between 6 and 10 mm appear clinically acceptable for IUI, but thresholds above this range require cautious interpretation.

## 7 OTHER DETERMINANTS OF INTRAUTERINE INSEMINATION SUCCESS

Several additional factors have been shown to significantly influence the success of IUI cycles and warrant further consideration. The method of ovarian stimulation plays a central role, with gonadotropins generally associated with higher pregnancy rates compared to clomiphene citrate or letrozole, albeit with an increased risk of multiple gestations.^30^ Moreover, the underlying cause of infertility impacts treatment outcomes women with unexplained infertility or ovulatory disorders typically show better responses to IUI compared to those with endometriosis or tubal factor infertility.^31^, ^76^ Ovarian reserve markers, such as anti‐Müllerian hormone antral follicle count, and baseline FSH, are also important predictors of ovarian responsiveness and overall treatment prognosis.^2^, ^32^ Racial and ethnic disparities in access to care, insurance coverage, and treatment success have been documented, highlighting the need for culturally sensitive and equitable fertility care.^33^, ^34^, ^78^, ^79^, ^80^ Expanding our understanding of these multifactorial influences is essential for personalizing treatment strategies and improving reproductive outcomes across diverse patient populations.

The duration of infertility, stimulation protocols used, cause of infertility, number of treatment cycles, timing of insemination, number of preovulatory follicles on the day of hCG administration, TMSC exceeding 10 million, and insemination count greater than 1 × 10^6^ with more than 4% normal sperm morphology are reported to affect the success rate of IUI.^2^ When considering the IUI methods used, the deliberate use of a modified slow‐release method for processed semen seems to notably increase clinical pregnancy rates following IUI with homologous semen.^77^ Further, studies reveal the importance of a personalized prognostic score to be applied to each couple to customize their expectations and improve the quality of counseling provided to prevent disappointment.^78^ In terms of psychological support, hope therapy for couples receiving IUI can positively affect their mood, increase fertility‐related attitudes, and increase pregnancy rates while reducing symptoms of depression, anxiety, and obsessive‐compulsive tendencies.^79^, ^80^, ^81^

Optimal IUI outcomes depend on hormonal and procedural factors. An estrogen concentration >500 pg/mL on the day of hCG administration^29^ and the use of gonadotropins with low‐dose protocols are associated with improved success rates, minimizing risks such as multiple pregnancies and ovarian hyperstimulation syndrome.^80^ Compared with clomiphene citrate, gonadotropins are associated with higher live birth rates (52%) (41%) (relative risk: 1.24, 95% confidence interval: 1.05–1.46).^82^ A recent systematic review reveals that among currently used IUI add‐ons, only vaginal luteal phase progesterone support shows probable benefit in improving live birth and ongoing pregnancy rates, while most other add‐on interventions, including endomentrial scratch, ovarian follicular phase stimulation, double insemination misoprostol, oxytocin, and bed rest after the IUI, lack sufficient evidence to support their routine clinical use and require further high‐quality research.^83^ Procedurally, the use of a soft catheter enhances outcomes, whereas the use of vaginal progesterone gel or micronized progesterone significantly improves clinical pregnancy rates.^82^ The timing between sperm preparation and IUI does not significantly affect success, even with waiting periods of up to 3 h.^83^, ^84^

**FIGURE 1:**
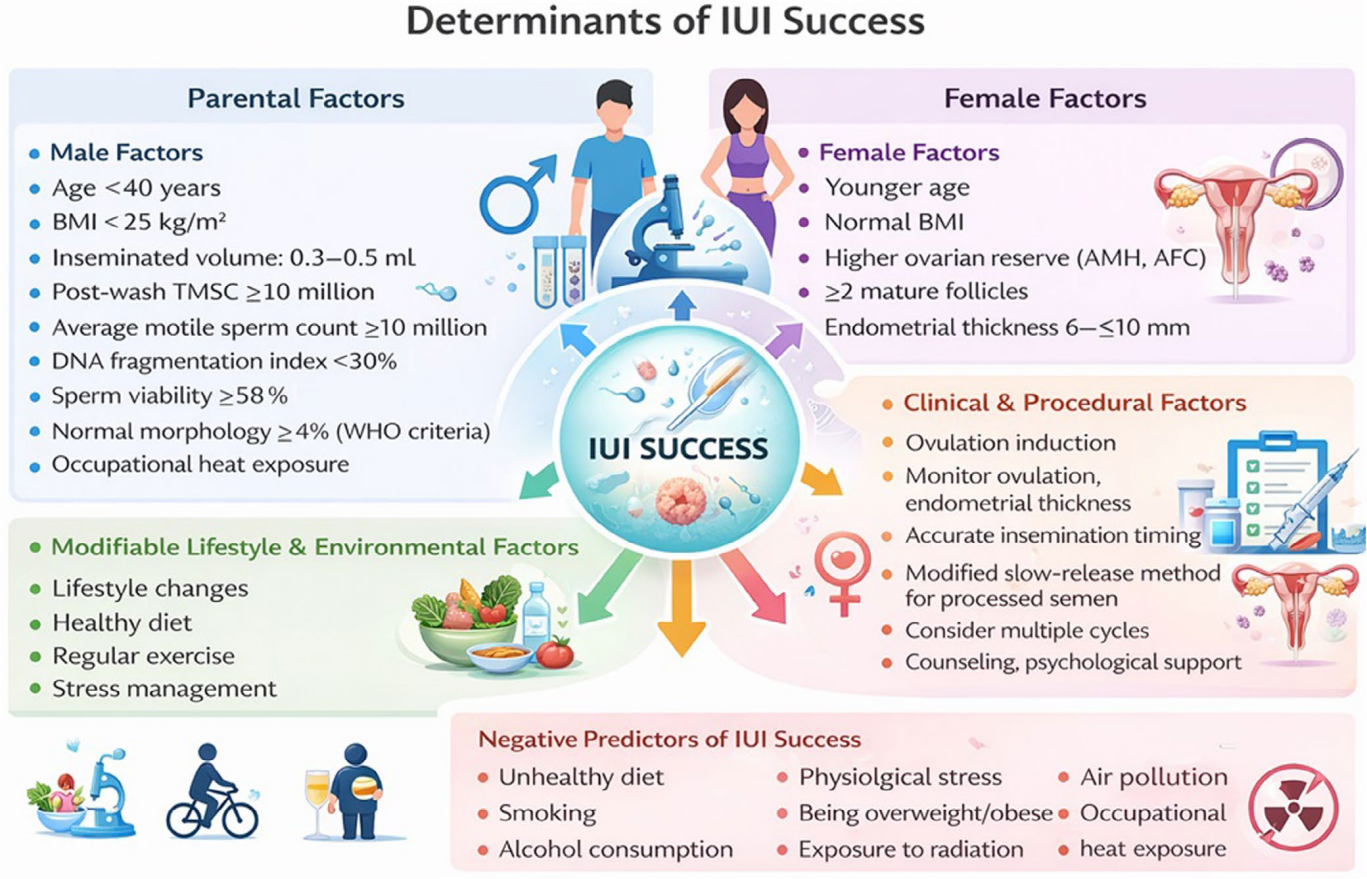
Determinants of the success rate of intrauterine insemination. BMI, body mass index; IUI, intrauterine insemination.

Figure 1 summarizes the key findings discussed in this review.

## 8 STUDY LIMITATIONS

This review has several limitations. The included studies varied widely in design, sample size, and IUI protocols, contributing to potential heterogeneity. Most were retrospective, limiting control over confounding factors. Differences in semen analysis methods and reporting of key parameters, such as motility and morphology, could have influenced comparability. Importantly, no formal risk‐of‐bias assessment was conducted, and the possibility of publication bias and exclusion of non‐English studies cannot be ruled out. Heterogeneity in infertility diagnosis categories (e.g., unexplained infertility, endometriosis, and male factor) also limits direct comparability across studies, as do differences in reporting at the cycle level versus couple level. Standardized, prospective studies are needed to validate these findings and refine clinical thresholds for IUI success.

## 9 CONCLUSION

The success of IUI is governed by the interplay of modifiable and clinical determinants. Optimizing maternal BMI, nutritional status (including adequate folate, zinc, selenium, and antioxidant vitamins), and psychological well‐being, alongside cessation of smoking and alcohol use, might improve treatment outcomes. Clinically, achieving an endometrial thickness of 6–10 mm, attaining a post‐wash TMSC ≥5–10 million, and using gonadotropin‐based stimulation protocols are associated with higher pregnancy rates. Integrating individualized counseling, lifestyle modification, and precise clinical monitoring should, therefore, form a central component of fertility management. Future research should focus on developing predictive models that combine biological, behavioral, and procedural determinants to refine personalized, evidence‐informed, and cost‐effective IUI care.

## AUTHOR CONTRIBUTIONS

Kasuni Akalanka solely conceived and designed the study, conducted the literature search and data analysis, interpreted the findings, and drafted and revised the manuscript. The author approved the final version for publication and is accountable for all aspects of the work.

## FUNDING INFORMATION

This research did not receive any specific grant from funding agencies in the public, commercial, or not‐for‐profit sectors.

## CONFLICT OF INTEREST STATEMENT

I have no conflicts of interests to declare.

## DECLARATION

Generative AI (ChatGPT, GPT‐5) was used solely for language refinement and readability enhancement of the manuscript. No AI tools were used for data extraction, statistical analysis, study selection, interpretation of findings, or scientific content generation.

## Data Availability

This manuscript is based on previously published studies; no new data were generated or analyzed.

## References

[1] Steures P , van der Steeg JW , Hompes PGA , van der Veen F , Mol BWJ . Intrauterine insemination in The Netherlands. Reprod Biomed Online. 2007;14:110‐116. 17207344 10.1016/s1472-6483(10)60772-9

[2] Allahbadia GN . Intrauterine insemination: fundamentals revisited. J Obstet Gynaecol India. 2017;67(6):385‐392. 29162950 10.1007/s13224-017-1060-x PMC5676579

[3] Cohlen BJ . Should we continue performing intrauterine inseminations in the year 2004? Gynecol Obstet Investig. 2005;59(1):3‐13. 15334020 10.1159/000080492

[4] Ramsay JE , Ferrell WR , Crawford L , Michael Wallace A , Greer IA , Sattar N . Maternal obesity is associated with dysregulation of metabolic, vascular, and inflammatory pathways. J Clin Endocrinol Metab. 2002;87:4231‐4237. 12213876 10.1210/jc.2002-020311

[5] Martínez‐Bermejo E , Luque‐Ramírez M , Escobar‐Morreale HF . Obesity and the polycystic ovary syndrome. Minerva Endocrinol. 2007;32:129‐140. 17912153

[6] Wang JX , Warnes GW , Davies MJ , Norman RJ . Overweight infertile patients have a higher fecundity than normal‐weight women undergoing controlled ovarian hyperstimulation with intrauterine insemination [6]. Fertil Steril. 2004;81(6):1710‐1712. 15193505 10.1016/j.fertnstert.2003.10.037

[7] Dodson WC , Kunselman AR , Legro RS . Association of obesity with treatment outcomes in ovulatory infertile women undergoing superovulation and intrauterine insemination. Fertil Steril. 2006;86:642‐646. 16782095 10.1016/j.fertnstert.2006.01.040

[8] Isa AM , Abu‐Rafea B , Alasiri SA , Binsaleh S , Ismail KH , Vilos GA . Age, body mass index, and number of previous trials: are they prognosticators of intrauterine‐insemination for infertility treatment? Int J Fertil Steril. 2014;8:255‐260. 25379153 PMC4221511

[9] Wang LT , Wang CX , Sun HL , et al. Effect of BMI on blood value of patients on HCG day with IUI treatment. BMC Womens Health. 2020;20(1):1‐8. 32410606 10.1186/s12905-020-00963-1 PMC7227362

[10] Whynott RM , Summers KM , Van Voorhis BJ , Mejia RB . Effect of body mass index on intrauterine insemination cycle success. Fertil Steril. 2021;115:221‐228. 33070966 10.1016/j.fertnstert.2020.07.003

[11] Starosta A , Gordon CE , Hornstein MD . Predictive factors for intrauterine insemination outcomes: a review. Fertil Res Pract. 2020;6(1):1‐11. 33308319 10.1186/s40738-020-00092-1 PMC7731622

[12] Yavuz A , Demirci O , Sözen H , Uludoǧan M . Predictive factors influencing pregnancy rates after intrauterine insemination. Iran J Reprod Med. 2013;11(3):227‐234. 24639750 PMC3943223

[13] Pandey S , Pandey S , Maheshwari A , Bhattacharya S . The impact of female obesity on the outcome of fertility treatment. J Hum Reprod Sci. 2010;3(2):62‐67. 21209748 10.4103/0974-1208.69332 PMC2970793

[14] Huyghe S , Verest ATA , Ombel W . Influence of BMI and smoking on IUI outcome with partner and donor sperm. Facts Views Vis Obgyn. 2017;9(2):93‐100. 29209485 PMC5707778

[15] Kohil A , Chouliaras S , Alabduljabbar S , et al. Female infertility and diet, is there a role for a personalized nutritional approach in assisted reproductive technologies? A narrative review. Front Nutr. 2022;9:927972. 35938101 10.3389/fnut.2022.927972 PMC9353397

[16] Cheah Y , Yang W . Functions of essential nutrition for high quality spermatogenesis. Adv Biosci Biotechnol. 2011;2(4):182‐197.

[17] Yilmaz N , Ersoy E , Tokmak A , Sargin A . Do serum vitamin D levels have any effect on intrauterine insemination success? Int J Fertil Steril. 2018;12(2):164‐168. 29707935 10.22074/ijfs.2018.5256 PMC5936616

[18] Montanaro Gauci M , Kruger TF , Coetzee K , Smith K , Van Der Merwe JP , Lombard CJ . Stepwise regression analysis to study male and female factors impacting on pregnancy rate in an intrauterine insemination programme. Andrologia. 2001;33:135‐141. 11380328 10.1046/j.1439-0272.2001.00428.x

[19] Farhi J , Orvieto R . Influence of smoking on outcome of COH and IUI in subfertile couples. J Assist Reprod Genet. 2009;26(7):421‐424. 19813098 10.1007/s10815-009-9330-x PMC2758945

[20] Niederberger C . Re: predictive value of different covariates influencing pregnancy rate following intrauterine insemination with homologous semen: a prospective cohort study. J Urol. 2017;198:1196. 10.1016/j.juro.2017.09.021 29144921

[21] Barbieri RL , McShane PM , Ryan KJ . Constituents of cigarette smoke inhibit human granulosa cell aromatase. Fertil Steril. 1986;46(2):232‐236. 3732529

[22] Huang H , Hansen KR , Factor‐Litvak P , et al. Predictors of pregnancy and live birth after insemination in couples with unexplained or male‐factor infertility. Fertil Steril. 2012;97:959‐967.e5. 22270557 10.1016/j.fertnstert.2012.01.090 PMC3319287

[23] Hansen KR , He ALW , Styer AK , et al. Predictors of pregnancy and live‐birth in couples with unexplained infertility after ovarian stimulation–intrauterine insemination. Fertil Steril. 2016;105:1575‐1583.e2. 26949110 10.1016/j.fertnstert.2016.02.020 PMC4893990

[24] Sirait B , Wiweko B , Jusuf AA , Handayani N , Aubry DA , Muharam R . Potential use of immature oocyte to improve fertility preservation outcome: a narrative review. J Hum Reprod Sci. 2022;15(1):3‐11. 35494192 10.4103/jhrs.jhrs_112_21 PMC9053342

[25] Thijssen A , Creemers A , Van der Elst W , et al. Predictive value of different covariates influencing pregnancy rate following intrauterine insemination with homologous semen: a prospective cohort study. Reprod Biomed Online. 2017;34(5):463‐472. 28285953 10.1016/j.rbmo.2017.01.016

[26] Lyngsø J , Bay B , Ingerslev HJ , Kesmodel US . Low‐to‐moderate alcohol consumption and success in fertility treatment: a Danish cohort study. Hum Reprod. 2019;34(7):1334‐1344. 31241750 10.1093/humrep/dez050

[27] Kan O , Gorkem U , Baser E , Alkilic A . Impact of anxiety levels on difficulty of intrauterine insemination and treatment outcomes. J Obstet Gynaecol Res. 2020;46:2059‐2065. 32715595 10.1111/jog.14398

[28] Syam HH , Madjid TH , Effendi JS , Djuwantono T , Permadi W , Fatma ZH . Anxiety effect in the success rate of intrauterine insemination (IUI) and in vitro fertilization (IVF). OALib. 2017;04:1‐10.

[29] Merviel P , Heraud MH , Grenier N , Lourdel E , Sanguinet P , Copin H . Predictive factors for pregnancy after intrauterine insemination (IUI): an analysis of 1038 cycles and a review of the literature. Fertil Steril. 2010;93:79‐88. 18996517 10.1016/j.fertnstert.2008.09.058

[30] Yousefi B , Azargon A . Predictive factors of intrauterine insemination success of women with infertility over 10 years. J Pak Med Assoc. 2011;61(2):165‐168. 21375168

[31] Esmailzadeh S , Faramarzi M . Endometrial thickness and pregnancy outcome after intrauterine insemination. Fertil Steril. 2007;88:432‐437. 17434500 10.1016/j.fertnstert.2006.12.010

[32] Patel AP , Patel MS , Shah SR , Jani SK . Predictive factors for pregnancy after intrauterine insemination: a retrospective study of factors affecting outcome. J South Asian Fed Obstet Gynaecol. 2016;8(2):140‐144.

[33] Patsama V , Supatchaya S , Khanitta T , Pornprom P , Chirawattana MSR . The influence of women age and successfulness of intrauterine insemination (IUI) cycles. J Med Assoc Thai. 2015;98(9):833‐838. 26591391

[34] Campana A , Sakkas D , Stalberg A , et al. Intrauterine insemination: evaluation of the results according to the woman's age, sperm quality, total sperm count per insemination and life table analysis. Hum Reprod. 1996;11(4):732‐736. 8671318 10.1093/oxfordjournals.humrep.a019244

[35] Hendin BN , Falcone T , Hallak J , et al. The effect of patient and semen characteristics on live birth rates following intrauterine insemination: a retrospective study. J Assist Reprod Genet. 2000;17:245‐252. 10976410 10.1023/A:1009402214820 PMC3455202

[36] Nuojua‐Huttunen S , Tomas C , Bloigu R , Tuomivaara L , Martikainen H . Intrauterine insemination treatment in subfertility: an analysis of factors affecting outcome. Hum Reprod. 1999;14(3):698‐703. 10221698 10.1093/humrep/14.3.698

[37] Sahakyan M , Harlow BL , Hornstein MD . Influence of age, diagnosis, and cycle number on pregnancy rates with gonadotropin‐induced controlled ovarian hyperstimulation and intrauterine insemination. Fertil Steril. 1999;72(3):500‐504. 10519623 10.1016/s0015-0282(99)00300-3

[38] Stone BA , Vargyas JM , Ringlet GE , et al. Determinants of the outcome of intrauterine insemination: analysis of outcomes of 9963 consecutive cycles. Am J Obstet Gynecol. 1999;180:1522‐1534. 10368500 10.1016/s0002-9378(99)70048-7

[39] Astolfi P , De Pasquale A , Zonta LA . Paternal age and preterm birth in Italy, 1990 to 1998. Epidemiology. 2006;17:218‐221. 16477263 10.1097/01.ede.0000197053.61176.f4

[40] Slama R , Bouyer J , Windham G , Fenster L , Werwatz A , Swan SH . Influence of paternal age on the risk of spontaneous abortion. Am J Epidemiol. 2005;161(9):816‐823. 15840613 10.1093/aje/kwi097

[41] Reichenberg A , Gross R , Weiser M , et al. Advancing paternal age and autism. Arch Gen Psychiatry. 2006;63(9):1026‐1032. 16953005 10.1001/archpsyc.63.9.1026

[42] Ford WCL , North K , Taylor H , Farrow A , Hull MGR , Golding J . Increasing paternal age is associated with delayed conception in a large population of fertile couples: evidence for declining fecundity in older men. Hum Reprod. 2000;15(8):1703‐1708. 10920089 10.1093/humrep/15.8.1703

[43] Bellver J , Garrido N , Remohí J , Pellicer A , Meseguer M . Influence of paternal age on assisted reproduction outcome. Reprod Biomed Online. 2008;17(5):595‐604. 18983742 10.1016/s1472-6483(10)60305-7

[44] Mathieu C , Ecochard R , Bied V , Lornage J , Czyba JC . Andrology: cumulative conception rate following intrauterine artificial insemination with husband's spermatozoa: influence of husband's age. Hum Reprod. 1995;10:1090‐1097. 7657747 10.1093/oxfordjournals.humrep.a136100

[45] Kumar M , Kumar R . Evaluation of male Factor infertility. AOGD Bull. 2019;19(2):8‐11.

[46] Ok EK , Doğan OE , Okyay RE , Gülekli B . The effect of postwash total progressive motile sperm count and semen volume on pregnancy outcomes in intrauterine insemination cycles: a retrospective study. J Turk Ger Gynecol Assoc. 2013;14(3):142‐145. 24592093 10.5152/jtgga.2013.52280 PMC3928411

[47] Kuriya A , Agbo C , Dahan MH . Do pregnancy rates differ with intrauterine insemination when different combinations of semen analysis parameters are abnormal? J Turk German Gynecol Assoc. 2018;19:57‐64. 10.4274/jtgga.2017.0082 PMC5994814 29553043

[48] Van Voorhis BJ , Barnett M , Sparks AET , Syrop CH , Rosenthal G , Dawson J . Effect of the total motile sperm count on the efficacy and cost‐effectiveness of intrauterine insemination and in vitro fertilization. Fertil Steril. 2001;75(4):661‐668. 11287015 10.1016/s0015-0282(00)01783-0

[49] Nikbakht R , Saharkhiz N . The influence of sperm morphology, total motile sperm count of semen and the number of motile sperm inseminated in sperm samples on the success of intrauterine insemination. Int J Fertil Steril. 2011;5:168‐173. 25101161 PMC4122832

[50] Cohlen BJ , Velde ER , Van Kooij RJ , Looman CWN , Habbema JDF . Controlled ovarian hyperstimulation and intrauterine insemination for treating male subfertility: a controlled study. Hum Reprod. 1998;13(6):1553‐1558. 9688391 10.1093/humrep/13.6.1553

[51] Gubert PG , Pudwell J , Van Vugt D , Reid RL , Velez MP . Number of motile spermatozoa inseminated and pregnancy outcomes in intrauterine insemination. Fertil Res Pract. 2019;5(1):1‐9. 31508237 10.1186/s40738-019-0062-z PMC6720098

[52] Mohammadi F , Mehdinia Z , Ghasemi S , et al. Relationship between sperm parameters and clinical outcomes of intra uterine insemination (IUI). Caspian J Intern Med. 2021;12(1):70‐76. 33680401 10.22088/cjim.12.1.70 PMC7919170

[53] Mollaahmadi L , Keramat A , Ghiasi A , Hashemzadeh M . The relationship between semen parameters in processed and unprocessed semen with intrauterine insemination success rates. J Turk Ger Gynecol Assoc. 2019;20:1‐7. 30222125 10.4274/jtgga.galenos.2018.2018.0089 PMC6501869

[54] Paulmyer‐Lacroix O , Mollé L , Noizet A , et al. Paulmyer‐Lacroix O, Mollé L, Noizet a, Guérin a, Mollar M, Gamerre M, Grillo JM. Inséminations intrautérines avec sperme du conjoint (IIU‐AC): conclusions de 5 ans d'expérience [intrauterine insemination with the husband's sperm: conclusions of five years experience] (French). Contracept Fertil Sex. 1998;26(4):300‐306. 9622965

[55] Dinelli L , Courbière B , Achard V , et al. Prognosis factors of pregnancy after intrauterine insemination with the husband's sperm: conclusions of an analysis of 2,019 cycles. Fertil Steril. 2014;101(4):994‐1000. 24534285 10.1016/j.fertnstert.2014.01.009

[56] Mankus EB , Holden AE , Seeker PM , et al. Prewash total motile count is a poor predictor of live birth in intrauterine insemination cycles. Fertil Steril. 2019;111(4):708‐713. 30929730 10.1016/j.fertnstert.2018.12.025 PMC6446576

[57] Dickey RP , Pyrzak R , Lu PY , Taylor SN , Rye PH . Comparison of the sperm quality necessary for successful intrauterine insemination with World Health Organization threshold values for normal sperm. Fertil Steril. 1999;71:684‐689. 10202879 10.1016/s0015-0282(98)00519-6

[58] Miller DC , Hollenbeck BK , Smith GD , et al. Processed total motile sperm count correlates with pregnancy outcome after intrauterine insemination. Urology. 2002;60(3):497‐501. 12350496 10.1016/s0090-4295(02)01773-9

[59] Huang HY , Lee CL , Lai YM , et al. The impact of the total motile sperm count on the success of intrauterine insemination with husband's spermatozoa. J Assist Reprod Genet. 1996;13:56‐63. 8825169 10.1007/BF02068871

[60] Ombelet W , Vandeput H , de Van Putte G , et al. Intrauterine insemination after ovarian stimulation with clomiphene citrate: predictive potential of inseminating motile count and sperm morphology. Hum Reprod. 1997;12(7):1458‐1463. 9262278 10.1093/humrep/12.7.1458

[61] Madbouly K , Isa A , Habous M , Almannie R , Abu‐Rafea B , Binsaleh S . Postwash total motile sperm count: should it be included as a standard male infertility work up. Can J Urol. 2017;24(3):8847‐8852. 28646941

[62] Zhang E , Tao X , Xing W , Cai L , Zhang B . Effect of sperm count on success of intrauterine insemination in couples diagnosed with male Factor infertility. Mater Soc. 2014;26:321. 10.5455/msm.2014.26.321-323 PMC4272842 25568631

[63] Findeklee S , Radosa JC , Radosa MP , Hammadeh ME . Correlation between total sperm count and sperm motility and pregnancy rate in couples undergoing intrauterine insemination. Sci Rep. 2020;10:7555. 32371917 10.1038/s41598-020-64578-0 PMC7200727

[64] Le MT , Nguyen TAT , Nguyen HTT , et al. Does sperm DNA fragmentation correlate with semen parameters? Reprod Med Biol. 2019;18:390‐396. 31607800 10.1002/rmb2.12297 PMC6780033

[65] Yang H , Li G , Jin H , Guo Y , Sun Y . The effect of sperm DNA fragmentation index on assisted reproductive technology outcomes and its relationship with semen parameters and lifestyle. Transl Androl Urol. 2019;8:356‐365. 31555559 10.21037/tau.2019.06.22 PMC6732090

[66] Bungum M , Humaidan P , Axmon A , et al. Sperm DNA integrity assessment in prediction of assisted reproduction technology outcome. Hum Reprod. 2007;22:174‐179. 16921163 10.1093/humrep/del326

[67] Marcello S , JensP B , Henrik IH , Henrik AK , Eugenia C , Giorgio L . Sperm chromatin damage impairs human fertility—fertility and sterility. 2000;73(1):43‐50. 10.1016/s0015-0282(99)00462-8 10632410

[68] Evenson DP , Wixon R . Data analysis of two in vivo fertility studies using Sperm Chromatin Structure Assay–derived DNA fragmentation index vs. pregnancy outcome. Fertil Steril. 2008;90(4):1229‐1231. 18191126 10.1016/j.fertnstert.2007.10.066

[69] Duran EH , Morshedi M , Taylor S , Oehninger S . Sperm DNA quality predicts intrauterine insemination outcome: a prospective cohort study. Hum Reprod. 2002;17(12):3122‐3128. 12456611 10.1093/humrep/17.12.3122

[70] Pacey AA . Environmental and lifestyle factors associated with sperm DNA damage. Hum Fertil (Camb). 2010;13:189‐193. 21117927 10.3109/14647273.2010.531883

[71] Van Rumste MME , Custers IM , Van Der Veen F , Van Wely M , Evers JLH , Mol BWJ . The influence of the number of follicles on pregnancy rates in intrauterine insemination with ovarian stimulation: a meta‐analysis. Hum Reprod Update. 2008;14(6):563‐570. 18687698 10.1093/humupd/dmn034

[72] Ghaffari F , Jahanian Sadatmahalleh S , Akhoond MR , Eftekhari Yazdi P , Zolfaghari Z . Evaluating the effective factors in pregnancy after intrauterine insemination: a retrospective study. Int J Fertil Steril. 2015;9:300‐308. 26644852 10.22074/ijfs.2015.4544 PMC4671382

[73] Weiss NS , Van Vliet MN , Limpens J , et al. Endometrial thickness in women undergoing IUI with ovarian stimulation. How thick is too thin? A systematic review and meta‐analysis. Hum Reprod. 2017;32(5):1009‐1018. 28333207 10.1093/humrep/dex035

[74] Danhof NA , van Eekelen R , Repping S , et al. Endometrial thickness as a biomarker for ongoing pregnancy in IUI for unexplained subfertility: a secondary analysis. Hum Reprod Open. 2020;2020(1):1‐6. 10.1093/hropen/hoz024 PMC6946937 31934648

[75] Habibzadeh V , Mahani SNN , Kamyab H . The correlation of factors affecting the endometrial thickness with pregnancy outcome in the IUI cycles. Iran J Reprod Med. 2011;9(1):41‐46. 25356081 PMC4212145

[76] Takasaki A , Tamura H , Miwa I , Taketani T , Shimamura K , Sugino N . Endometrial growth and uterine blood flow: a pilot study for improving endometrial thickness in the patients with a thin endometrium. Fertil Steril. 2010;93(6):1851‐1858. 19200982 10.1016/j.fertnstert.2008.12.062

[77] Ombelet W , Van der Auwera I , Bijnens H , et al. Improving IUI success by performing modified slow‐release insemination and a patient‐centred approach in an insemination programme with partner semen: a prospective cohort study. Facts Views Vis Obgyn. 2021;13(4):359‐367. 35026097 10.52054/FVVO.13.4.045 PMC9148711

[78] Huniadi A , Bimbo‐Szuhai E , Botea M , et al. Fertility predictors in intrauterine insemination (IUI). J Pers Med. 2023;13(3):395. 36983577 10.3390/jpm13030395 PMC10058138

[79] Zhu H , Xu S , Wang M , Shang Y , Wei C , Fu J . Effect of hope therapy on fertility stress and pregnancy rate in infertile patients undergoing intrauterine insemination. Am J Transl Res. 2022;14(6):4363‐4371. 35836885 PMC9274543

[80] Gadson AK , Sauerbrun‐Cutler MT , Eaton JL . Racial disparities in fertility care: a narrative review of challenges in the utilization of fertility preservation and ART in minority populations. J Clin Med. 2024;13(4):1060. doi:10.3390/jcm13041060 38398373 PMC10889491

[81] Dias CMF , Vitorino GBT , Furlan SMP , et al. Intrauterine insemination: prognostic factors. JBRA Assist Reprod. 2024;28(2):254‐262. doi:10.5935/1518-0557.20240017 38546118 PMC11152422

[82] Oktem M , Altinkaya SO , Yilmaz SA , et al. Effect of luteal phase support after ovulation induction and intrauterine insemination. Gynecol Endocrinol. 2014;30(12):909‐912. doi:10.3109/09513590.2014.947567 25102275

[83] Chronopoulou E , Gaetano‐Gil A , Shaikh S , et al. Optimizing intrauterine insemination: a systematic review and meta‐analysis of the effectiveness and safety of clinical treatment add‐ons. Acta Obstet Gynecol Scand. 2024;103(10):1919‐1932. doi:10.1111/aogs.14858 38961556 PMC11426219

[84] Stein A , Altman E , Rotlevi M , et al. Does the time interval from the end of sperm processing to intrauterine insemination (lab‐to‐uterus time) affect treatment outcome? Andrology. 2021;9(6):1859‐1863. doi:10.1111/andr.13079 34245222

